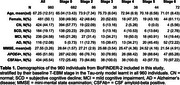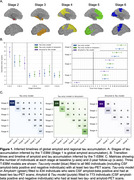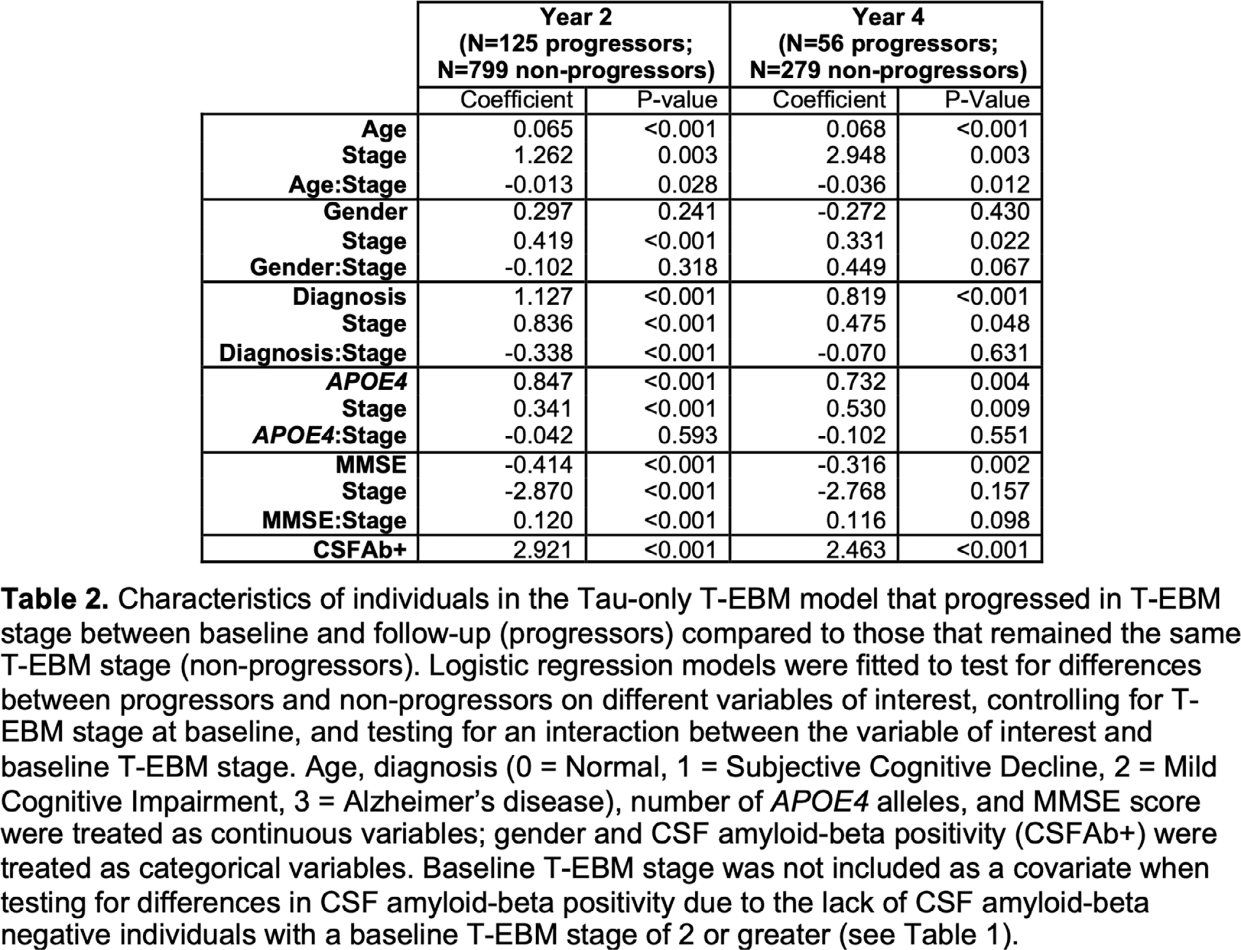# Disease timeline modelling of amyloid and tau PET reveals dynamic timescales of amyloid and tau accumulation in Alzheimer’s disease

**DOI:** 10.1002/alz70862_110838

**Published:** 2025-12-23

**Authors:** Alexandra L Young, Peter A Wijeratne, Leon M. Aksman, Alexa Pichet Binette, Olof Strandberg, Neil P Oxtoby, Andre Altmann, Daniel C Alexander, Erik Stomrud, Sebastian Palmqvist, Niklas Mattsson‐Carlgren, Jacob W. Vogel, Oskar Hansson

**Affiliations:** ^1^ UCL Hawkes Institute and Department of Computer Science, University College London, London UK; ^2^ Sussex AI Centre, Department of Informatics, University of Sussex, Brighton UK; ^3^ Mark and Mary Stevens Neuroimaging and Informatics Institute, Keck School of Medicine, Los Angeles, CA USA; ^4^ Department of Physiology and Pharmacology, Université de Montréal, Montréal, QC Canada; ^5^ Centre de Recherche de l’Institut Universitaire de Gériatrie de Montréal, Montréal, QC Canada; ^6^ Clinical Memory Research Unit, Department of Clinical Sciences Malmö, Faculty of Medicine, Lund University, Lund Sweden; ^7^ Memory Clinic, Skåne University Hospital, Malmö Sweden; ^8^ UCL Hawkes Institute and Department of Medical Physics and Biomedical Engineering, University College London, London UK; ^9^ Memory Clinic, Skåne University Hospital, Malmö, Skåne Sweden; ^10^ Wallenberg Center for Molecular Medicine, Lund University, Lund Sweden; ^11^ Department of Clinical Sciences Malmö, Faculty of Medicine, SciLifeLab, Lund University, Lund Sweden; ^12^ Clinical Memory Research Unit, Lund University, Malmö, Skåne Sweden

## Abstract

**Background:**

Amyloid and tau accumulation in Alzheimer’s disease is known to be dynamic, with expected rates of accumulation varying depending on disease stage. Establishing the precise timeline of amyloid and tau accumulation and quantifying their dynamic progression is important for identifying an optimal intervention window and predicting treatment response.

**Method:**

960 individuals were selected from the Swedish BioFINDER‐2 study with at least two tau‐PET scans (Table 1; follow‐ups were at 1 year (*N* = 66), 2 years (*N* = 924), 4 years (*N* = 335); 6 years (*N* = 60)). Two intersecting data subsets were selected: 773 individuals having at least two amyloid‐PET scans for estimating amyloid duration, and 434 CSF‐amyloid‐positive individuals for comparison with timelines across the whole population. Regional tau‐PET SUVR abnormality was computed in five established data‐driven regions using mixture modelling. A novel explicit‐duration version of the temporal event‐based model (T‐EBM) was used to determine the order and timeline of global amyloid‐PET and regional tau‐PET abnormality. The explicit duration approach accounts for censoring of an individual’s first and last visit and handles arbitrary time intervals.

**Result:**

The T‐EBM inferred that tau accumulates in a Braak‐like pattern (Figure 1a), estimating an average timeline of global amyloid and regional tau accumulation (Figure 1b) of around 20 years. Progression from stage 1 (amyloid) to stage 2 (entorhinal tau) was estimated to take 8 years on average, from stage 2 to stage 3 (temporal lobe tau) 5.5 years, and 2‐3 years between each subsequent stage. The timeline was consistent in amyloid‐positive individuals (most amyloid‐negative individuals were stage 0 and did not influence the timeline). Figure 1c shows the number of individuals progressing between stages at follow‐up. Individuals who progressed in stage (progressors) were older, had more advanced symptoms (diagnosis), more *APOE4* alleles, worse MMSE scores, and were more frequently amyloid‐positive compared to non‐progressors (Table 2).

**Conclusion:**

Amyloid accumulates slowly, after which tau spreads from the entorhinal cortex to the temporal lobe, initially at a slower pace before accelerating to a faster rate across the cortex. This data indicates that slower rates of accumulation would be expected at earlier stages. Work is ongoing validating these timelines in additional datasets.